# Quarantine and Public Health

**Published:** 1881-01

**Authors:** 


QUARANTINE AND PUBLIC HEALTH. — As our readers are aware, two medical assemblies occurred in New Orleans in the early part of December, 1880.

One was the regular annual meeting of the American Public Health Association, and the other a Special Quarantine Convention. The former appears to have been well attended, and many papers and reports on important topics relating to public health and the propagation of disease, were read and discussed. The National Board of Health received a due share of attention and approval. On the closing day of the session an Advisory Council of the Board was appointed, consisting of one representative from each State. The representatives in this Advisory Council for the Northwestern States are: Dr. J. H. Rauch, of Illinois; Dr. J. F. Hibberd, of Indiana; Dr. W. S. Robertson, of Iowa; Dr. H. B. Baker, of Michigan; Dr. J. T. Reeve, of Wisconsin, and Dr. C. M. Hewett, of Minnesota.

The Quarantine Convention, which was also attended by many of the delegates to the Public Health Association, appears to have done little else than to discuss the question whether the quarantine regulations of the Mississippi Valley, as well as the seaports, should be under the supreme control of the National Board of Health, or should remain under the immediate control of the local boards of each State.

It is evident that the members of the Convention were pretty evenly divided in regard to this important subject, and its consideration ended in no decision in favor of either party. As a compromise, the following resolutions, offered by Hon. William R. Moore, of Memphis, were adopted, and the Convention adjourned. The resolutions are as follows:

*Resolved*, That the chair appoint two committees, to consist of seven members each, one committee to represent the Atlantic and Gulf States here represented, and one to represent the States of the Ohio and Mississippi Valleys, each committee to prepare a schedule of rules and regulations concerning those matters of quarantine sanitation which are common to the States of each region respectively, and which schedules shall be submitted to each of such States for ratification and adoption as a basis of action for the protection of the public health; no state to have more than one representative on either of said committees.

*Resolved*, That it is the duty of the general government to defray the expenses of all quarantine administration of this character which extends beyond the boundaries of a single State, and said committee are hereby authorized and instructed to take the necessary steps to secure adequate appropriations by Congress for this purpose; such appropriations to be disbursed and expended in accordance with the usual Treasury regulations concerning disbursements and expenditures.

*Resolved*, That the chair be authorized to announce the members of the committee at any time within the next ten days.

